# Viscosity iterative algorithm for the zero point of monotone mappings in Banach spaces

**DOI:** 10.1186/s13660-018-1845-1

**Published:** 2018-09-21

**Authors:** Yan Tang

**Affiliations:** College of Mathematics and Statistics, Chongqing Key Laboratory of Social Economy and Applied Statistics, Chongqing Technology and Business University, Chongqing, China

**Keywords:** 47H04, 46N10, 47H06, 47J25, Monotone mapping, Zero point, Viscosity approximation, Strong convergence

## Abstract

Inspired by the work of Zegeye (J. Math. Anal. Appl. 343:663–671, 2008) and the recent papers of Chidume et al. (Fixed Point Theory Appl. 2016:97, 2016; Br. J. Math. Comput. Sci. 18:1–14, 2016), we devise a viscosity iterative algorithm without involving the resolvent operator for approximating the zero of a monotone mapping in the setting of uniformly convex Banach spaces. Under concise parameter conditions we establish strong convergence of the proposed algorithm. Moreover, applications to constrained convex minimization problems and solution of Hammerstein integral equations are included. Finally, the performances and computational examples and a comparison with related algorithms are presented to illustrate the efficiency and applicability of our new algorithm.

## Introduction

Let *H* be a real inner product space. A map $A:D(A)\subset H\rightarrow2^{H}$ is called monotone if, for each $x, y\in D(A)$, the following inequality holds:

1.1 $$\begin{aligned} \langle\xi-\eta,x-y \rangle\geq0 \quad\mbox{for all }\xi \in Ax,\eta\in Ay. \end{aligned}$$

Interest in monotone mappings derives mainly from their significant numerous applications. For example, the classical convex optimization problem: let $h:H\rightarrow\mathbb{R}\cup\{\infty \}$ be a proper convex, lower semicontinuous (*l.s.c.*) function. The sub-differential of *h* at $x\in H$ is defined by $\partial h:H\rightarrow2^{H}$

$$\begin{aligned} \partial h(x)=\bigl\{ x^{*}\in H:h(y)-h(x)\geq\bigl\langle y-x,x^{*}\bigr\rangle , \forall y\in H\bigr\} . \end{aligned}$$

Clearly, *∂h* is a monotone operator on *H*, and $0\in\partial h(x_{0})$ if and only if $x_{0}$ is a minimizer of *h*. In the case of setting $\partial h\equiv A$, solving the inclusion $0\in Au$, one obtains a minimizer of *h*.

In addition, the inclusion $0\in Au$ when *A* is a monotone map from a real Hilbert space to itself also appears in several systems, in particular, evolution systems:

$$\begin{aligned} \frac{du}{dt}+Au=0, \end{aligned}$$

where *A* is a monotone map. At an equilibrium state, $\frac {du}{dt}=0$, so that $Au=0$, the solution coincides with the equilibrium state of the dynamical system (see, e.g., Zarantonello [4], Minty [5], Kac̆urovskii [6], Chidume [7], Berinde [8], and others).

For solving the original problem of finding a solution of the inclusion $0\in Au$, Martinet [9] introduced the well-known iteration method as follows: for $n\in\mathbb{N}$, $\forall\lambda_{n}>0$, $x_{1} \in E$ and

$$ x_{n+1}=J_{\lambda_{n}}x_{n}, $$

where $J_{\lambda_{n}}=(I+\lambda_{n}A)^{-1}$ is the well-known Yosida resolvent operator, *A* is a monotone operator in Hilbert spaces.

This is a successful and powerful algorithm in finding a solution of the equation $0\in Au$ and after that, it was extended by many authors (see, e.g., Rockafellar [10], Chidume [11], Xu [12], Tang [13], Qin et al. [14]).

On the other hand, Browder [15] introduced an operator $T:H\rightarrow H$ by $T=I-A$ where *I* is the identity mapping on a Hilbert space *H*. The operator *T* is called pseudo-contractive and the zeros of monotone operator *A*, if they exist, correspond to the fixed points of *T*. Therefore the approximation of the solutions of $Au=0$ reduces to the approximation of the fixed points of a pseudo-contractive mapping.

Gradually, the notion of monotone mapping has been extended to real normed spaces. Let *E* be a real normed space with dual $E^{*}$. A map $J:E\rightarrow 2^{E^{*}}$, defined by

1.2 $$\begin{aligned} Jx:= \bigl\{ x^{*}\in E^{*}: \bigl\langle x,x^{*} \bigr\rangle = \Vert x \Vert \cdot \bigl\Vert x^{*} \bigr\Vert , \Vert x \Vert = \bigl\Vert x^{*} \bigr\Vert \bigr\} , \end{aligned}$$

is called the normalized duality map on *E*. Some properties of the normalized duality map can be obtained from Alber [16] and the references therein.

Since the normalized duality map *J* is the identity map *I* in Hilbert spaces, and so, under the idea of Browder [15], the approximating to solution of $0\in Au$ has been extended to normed spaces by numerous authors (see, for instance, Chidume [17, 18], Agarwal et al. [19], Reich [20], Diop [21], and the references therein), where *A* is a monotone mapping from *E* to itself.

Although the above results have better theoretical properties, such as, but not only, weak and strong convergence to a solution of the equation $0\in Au$, there are still some difficulties to overcome. For instance, the generalized technique of converting the zero of *A* into the fixed point of *T* in Browder [15] is not applicable since, in this case when *A* is monotone, *A* maps *E* into $E^{*}$. In addition, the resolvent technique in Martinet [9] is not convenient to use because one has to compute the inverse of $(I+\lambda A)$ at each step of the iteration process.

Hence, it is only natural to ask the following question.

### Question 1.1

Can we construct an algorithm without involving the resolvent operator to approximate a zero point of *A* in Banach spaces?

Motivated and inspired by the work of Martinet [9], Rockafellar [10], Zegeye [1], and Chidume et al. [2, 3], as well as Ibaraki and Takahashi [22], we wish to provide an affirmative answer to the question. Our contribution in the present work is a new viscosity iterative method for the solutions of the equation $0 \in AJu$, that is, $Ju\in A^{-1}(0)$, where $A: E^{*} \rightarrow{E}$ is a monotone operator defined on the dual of a Banach space *E* and $J:E\rightarrow E^{*}$ is the normalized duality map.

The outline of the paper is as follows. In Sect. 2, we collect definitions and results which are needed for our further analysis. In Sect. 3, our implicit and explicit algorithms without involving a resolvent operator are introduced and analyzed, the strong convergence to a zero of the composed mapping *AJ* under concise parameters conditions is obtained. In addition, the main result is applied to the convex optimization problems and the solution of Hammerstein equation. Finally, some numerical experiments and a comparison with related algorithms are given to illustrate the performances of our new algorithms.

## Preliminaries

In the sequel, we shall need the following definitions and results.

Let *E* be a uniformly convex Banach space and $E^{*}$ be its dual space, let the normalized duality map *J* on *E* be defined as (1.2). Then the following properties of the normalized duality map hold (see, e.g., Alber [16], Cioranescu [23], Xu and Roach [24], Xu [25], Zălinescu [26]):

(i) *J* is a monotone operator;
(ii) if *E* is smooth, then *J* is single-valued;
(iii) if *E* is reflexive, then *J* is onto;
(iv) if *E* is uniformly smooth, then *J* is uniformly continuous on bounded subsets of *E*.

The space *E* is said to be smooth if $\rho_{E}(\tau)>0$ for all $\tau >0$, and the space *E* is said to be uniformly smooth if $\lim_{\tau\rightarrow0^{+}}\frac{\rho_{E}(\tau)}{\tau}=0$, where $\rho _{E}(\tau)$ is defined by

$$\begin{aligned} \rho_{E}(\tau)=\sup\biggl\{ \frac{ \Vert x+y \Vert - \Vert x-y \Vert }{2}-1; \Vert x \Vert =1, \Vert y \Vert =\tau\biggr\} . \end{aligned}$$

Let $p>1$, the space *E* is said to be *p*-uniformly smooth if there exists a constant $c>0$ such that $\rho_{E}(\tau) \leq c \tau^{p}$, $\tau>0$. It is well known that every *p*-uniformly smooth Banach space is uniformly smooth. Furthermore, from Alber [16], we can get that if *E* is 2-uniformly smooth, then there exists a constant $L_{*}>0$ such that

$$\begin{aligned} \Vert Jx-Jy \Vert \leq L_{*} \Vert x-y \Vert ,\quad \forall x,y\in E. \end{aligned}$$

A mapping $A:D(A)\subset E\rightarrow E^{*}$ is said to be monotone on a Banach space *E* if, for each $x,y\in D(A)$, the following inequality holds:

$$\begin{aligned} \langle x-y,Ax-Ay\rangle\geq0. \end{aligned}$$

A mapping $A:D(A)\subset E\rightarrow E^{*}$ is said to be Lipschitz continuous if there exists $L>0$ such that, for each $x, y\in D(A)$, the following inequality holds:

$$\begin{aligned} \Vert Ax-Ay \Vert _{E^{*}}\leq L \Vert x-y \Vert _{E}. \end{aligned}$$

A mapping $f: E\rightarrow E$ is called contractive if there exists a constant $\rho\in(0,1)$ such that

$$\begin{aligned} \bigl\Vert f(x)-f(y) \bigr\Vert \leq\rho \Vert x-y \Vert , \quad \forall x,y \in E. \end{aligned}$$

Let *C* be a nonempty closed convex subset of a uniformly convex Banach space *E*. A Banach limit *μ* is a bounded linear functional on $l^{\infty}$ such that

$$ \inf\{x_{n};n\in\mathbb{N}\}\leq\mu(x)\leq\sup\{x_{n};n\in \mathbb{N}\}, \quad \forall x=\{x_{n}\}\in l^{\infty}, $$

and $\mu(x_{n})=\mu(x_{n+1})$ for all $\{x_{n}\} \in l^{\infty}$. Suppose that $\{x_{n}\}$ is a bounded sequence in *E*, then the real valued function *φ* on *E* defined by

2.1 $$ \varphi(y)=\mu \Vert x_{n}-y \Vert ^{2},\quad \forall y\in E, $$

is convex and continuous, and $\varphi(y)\rightarrow\infty$ as $\|y\|\rightarrow\infty$. If *E* is reflexive, there exists $z\in C$ such that $\varphi(z)=\min_{y\in C}\varphi(y)$ (see, e.g., Kamimura and Takahashi [27], Tan and Xu [28]), so we can define the set $C_{\min}$ by

2.2 $$\begin{aligned} C_{\min}=\Bigl\{ z\in C; \varphi(z)=\min_{y\in C} \varphi(y)\Bigr\} . \end{aligned}$$

It is easy to verify that $C_{\min}$ is a nonempty, bounded, closed, and convex subset of *E*. The following lemma was proved in Takahashi [29].

### Lemma 2.1

*Let* *α* *be a real number*, *and* $(x_{0},x_{1},\ldots)\in l^{\infty}$ *such that* $\mu(x_{n})\leq\alpha$ *for all Banach limits*. *If* $\limsup_{n\rightarrow\infty}(x_{n+1}-x_{n})\leq0$, *then* $\limsup_{n\rightarrow\infty}x_{n}\leq\alpha$.

### Lemma 2.2

(see, e.g., Tan and Xu [28], Osilike and Aniagbosor [30])

*Let* $\{a_{n}\}$ *be a sequence of nonnegative real numbers satisfying the following relation*:

$$\begin{aligned} a_{n+1} \leq(1-\theta_{n})a_{n}+ \sigma_{n}, \quad n\geq0, \end{aligned}$$

*where* $\{\theta_{n}\}$ *and* $\{\sigma_{n}\}$ *are real sequences such that*

(i) $\lim_{n\rightarrow\infty}\theta_{n}=0$, $\sum_{n=1}^{\infty}\theta_{n}=\infty$;
(ii) $\lim_{n\rightarrow\infty}\frac{\sigma_{n}}{\theta_{n}}\leq0$ *or* $\sum_{n=0}^{\infty}\sigma_{n}<\infty$.

*Then the sequence* $\{a_{n}\}$ *converges to* 0.

### Lemma 2.3

(see, e.g., Xu [12])

*Let* *E* *be a real Banach space with dual* $E^{*}$. *Let* $J:E\rightarrow E^{*}$ *be the normalized duality map*, *then for all* $x,y\in{E}$,

$$ \Vert x+y \Vert ^{2}\leq \Vert x \Vert ^{2}+2{\bigl\langle y,j(x+y)\bigr\rangle },\quad \forall{j(x+y)}\in{J(x+y)}. $$

### Lemma 2.4

(Zegeye [1])

*Let* *E* *be a uniformly convex and uniformly smooth Banach space*. *Assume that* $A:E^{*}\rightarrow E$ *is a maximal monotone mapping such that* $(AJ)^{-1}(0)\neq\emptyset$. *Then*, *for any* $u\in E$ *and* $t\in(0,1)$, *the path* $t\rightarrow x_{t} \in E$ *defined by*

2.3 $$ x_{t}=tu+(1-t) (I- AJ)x_{t}, $$

*converges strongly to an element* $z\in(AJ)^{-1}(0)$ *as* $t\rightarrow0$.

## Main results

We now show the strong convergence of our implicit and explicit algorithms.

### Theorem 3.1

*Let* *E* *be a uniformly convex and* 2-*uniformly smooth Banach space*. *Assume that* $A:E^{*}\rightarrow E$ *is an* *L*-*Lipschitz continuous monotone mapping such that* $(AJ)^{-1}(0) \neq\emptyset$ *and* $f:E\rightarrow E$ *is a contraction with coefficient* $\rho\in(0,1)$. *Then the path* $t\rightarrow x_{t} \in E$, *defined by*

3.1 $$ x_{t}=tf(x_{t})+(1-t) \bigl(I-\omega(t)AJ \bigr)x_{t}, $$

*converges strongly to an element* $z\in(AJ)^{-1}(0)$ *provided that* $\lim_{t\rightarrow0}\frac{\omega(t)}{t}=0$.

### Proof

Since *E* is 2-uniformly smooth, from Alber [16, 31], we have that *J* is $L_{*}$-Lipschitz continuous, noticing that *A* is *L*-Lipschitz continuous, therefore $I-AJ$ is Lipschitz continuous with constant $1+LL_{*}$.

First, we show that $x_{t}$ is well-defined. Since $\lim_{t\rightarrow0}\frac{\omega(t)}{t}=0$, for $\forall \varepsilon>0$, there exists $\delta>0$ such that, for all $t\in(0,\delta)$, the inequality $|\frac{\omega (t)}{t}|<\varepsilon$ holds.

Without loss of generality, we take $\varepsilon>0$ such that $\rho +\varepsilon LL_{*}=b<1$, where *b* is a positive constant. Define an operator $T_{t}$ as $T_{t}x=f(x)-(1-t)\frac{\omega(t)}{t}AJx$, for $\forall x, y\in E$, we can get

$$\begin{aligned} \Vert T_{t}x-T_{t}y \Vert =& \biggl\Vert f(x)-(1-t) \frac{\omega(t)}{t}AJx- f(y)+(1-t)\frac {\omega(t)}{t}AJy \biggr\Vert \\ =& \biggl\Vert f(x)-f(y)-(1-t)\frac{\omega(t)}{t} (AJx-AJy) \biggr\Vert \\ \leq& \bigl\Vert f(x)-f(y) \bigr\Vert + \biggl\vert (1-t) \frac{\omega(t)}{t} \biggr\vert \Vert AJx-AJy \Vert \\ \leq&(\rho+\varepsilon LL_{*}) \Vert x-y \Vert \\ =&b \Vert x-y \Vert , \end{aligned}$$

which means that $T_{t}$ is a contraction. Therefore, by the Banach contraction principle, there exists a unique fixed point of $T_{t}$ denoted by $x_{t}$. That is, $x_{t}=tf(x_{t})+(1-t)(I-\omega(t)AJ)x_{t}$, so $x_{t}$ is well-defined.

Next we shall show that $x_{t}$ is bounded as $\lim_{t\rightarrow0}\frac {\omega(t)}{t}=0$. For $x^{*}\in(AJ)^{-1}(0)$, we have the following estimation:

$$\begin{aligned} \bigl\Vert x_{t}-x^{*} \bigr\Vert =& \biggl\Vert f(x_{t})-x^{*}-(1-t)\frac{\omega(t)}{t}\bigl(AJx_{t}-AJx^{*} \bigr) \biggr\Vert \\ \leq& \bigl\Vert f(x_{t})-f\bigl(x^{*}\bigr) \bigr\Vert + \bigl\Vert f\bigl(x^{*}\bigr)-x^{*} \bigr\Vert +(1-t)\frac{\omega(t)}{t}LL_{*} \bigl\Vert x_{t}-x^{*} \bigr\Vert \\ \leq& (\rho+\varepsilon LL_{*}) \bigl\Vert x_{t}-x^{*} \bigr\Vert + \bigl\Vert f\bigl(x^{*}\bigr)-x^{*} \bigr\Vert , \end{aligned}$$

hence,

$$\begin{aligned} \bigl\Vert x_{t}-x^{*} \bigr\Vert \leq\frac{1}{1-b} \bigl\Vert f\bigl(x^{*}\bigr)-x^{*} \bigr\Vert ,\quad t\in(0,\delta), \end{aligned}$$

which means that $x_{t}$ is bounded as $t\rightarrow0$, therefore is $f(x_{t})$.

On the other hand, for arbitrary $u\in E$, (3.1) can be rewritten as

$$\begin{aligned} x_{t}=tu+(1-t) (I-AJ)x_{t}+t\bigl(f(x_{t})-u \bigr)+(1-t) \bigl(1-\omega(t)\bigr)AJx_{t}, \end{aligned}$$

hence,

$$\begin{aligned} (1-t) \bigl(1-\omega(t)\bigr)AJx_{t}=x_{t}-tu-(1-t) (I-AJ)x_{t}-t\bigl(f(x_{t})-u\bigr), \end{aligned}$$

which means that $x_{t}$ converges strongly to an element $z\in (AJ)^{-1}(0)$ as $\lim_{t\rightarrow0}\frac{\omega(t)}{t}=0$ according to Lemma 2.4. The proof is complete. □

For the rest of the paper, $\{\alpha_{n}\}$ and $\{\omega_{n}\}$ are real sequences in $(0,1)$ satisfying the following conditions:

(C1): $\lim_{n\rightarrow\infty}\alpha_{n}=0$, $\sum_{n=1}^{\infty}\alpha_{n}=\infty$; $\lim_{n\rightarrow\infty}\frac{\omega_{n}}{\alpha_{n}}=0$ and $\sum_{n=0}^{\infty}\omega_{n}<\infty$;
(C2): *f* is a piecewise function: $f(x^{*})=x^{*}$ if $x^{*}\in(AJ)^{-1}(0)$; otherwise $f(x^{*})$ is a contractive function with coefficient *ρ*.

### Theorem 3.2

*Let* *E* *be a uniformly convex and* 2-*uniformly smooth Banach space*. *Assume that* $A:E^{*}\rightarrow E$ *is an* *L*-*Lipschitz continuous monotone mapping such that* $C_{\min}\cap (AJ)^{-1}(0) \neq\emptyset$ *and* $f:E\rightarrow E$ *is a piecewise function defined as* (C2). *Then*, *for any* $x_{0}\in E$, *the sequence* $\{x_{n}\}$, *defined by*

3.2 $$ x_{n+1}=\alpha_{n}f(x_{n})+(1- \alpha_{n}) (I-\omega_{n}AJ)x_{n}, $$

*converges strongly to an element* $z\in(AJ)^{-1}(0)$.

### Proof

According to the definition of *f*, it is obvious that if $x_{n}\in (AJ)^{-1}(0) $ then we stop the iteration. Otherwise, we set $n:=n+1$ and return to iterative step (3.2).

The proof includes three steps.

*Step 1*: First we prove that $\{x_{n}\}$ is bounded. Since $\alpha _{n}\rightarrow0$ and $\lim_{n\rightarrow\infty}\frac{\omega_{n}}{\alpha _{n}}=0$ as $n\rightarrow\infty$, there exists $N_{0}>0$ such that $\alpha _{n}\leq\frac{1}{6}$, $\frac{\omega_{n}}{\alpha_{n}}\leq\frac{1}{6LL_{*}}$, $\forall n>N_{0}$. We take $x^{*}\in(AJ)^{-1}(0)$ or $Jx^{*}\in A^{-1}(0)$. Let $r>0$ be sufficiently large such that $x_{N_{0}}\in B_{r}(x^{*})$ and $f(x_{N_{0}})\in B_{\frac{r}{6}}(x^{*})$.

We show that $\{x_{n}\}$ belongs to $B:=\overline{B_{r}(x^{*})}$ for all integers $n\geq N_{0}$. First, it is clear by construction that $x_{N_{0}}\in B$. Assuming now that, for an arbitrary $n>N_{0}$, $x_{n}\in B$, we prove that $x_{n+1} \in B$.

If $x_{n+1}$ does not belong to *B*, then we have $\|x_{n+1}-x^{*}\|>r$. From the recurrence (3.2) we obtain that

$$\begin{aligned} x_{n+1}-x_{n}=\alpha_{n}f(x_{n})+(1- \alpha_{n}) (I-\omega_{n}AJ)x_{n}-x_{n}. \end{aligned}$$

Thus,

3.3 $$\begin{aligned} x_{n+1}-x_{n}=\alpha_{n}\bigl(f(x_{n})-x_{n} \bigr)-(1-\alpha_{n})\omega_{n}AJx_{n}. \end{aligned}$$

Therefore, from (3.3) and Lemma 2.3 and the fact that $x_{n+1}-x^{*}=x_{n+1}-x_{n}+x_{n}-x^{*}$,

$$\begin{aligned} \bigl\Vert x_{n+1}-x^{*} \bigr\Vert ^{2} =& \bigl\Vert x_{n+1}-x_{n}+x_{n}-x^{*} \bigr\Vert ^{2} \\ \leq& \bigl\Vert x_{n}-x^{*} \bigr\Vert ^{2}+2\bigl\langle x_{n+1}-x_{n}, j\bigl(x_{n+1}-x^{*}\bigr)\bigr\rangle \\ =& \bigl\Vert x_{n}-x^{*} \bigr\Vert ^{2}+2\bigl\langle \alpha_{n}\bigl(f(x_{n})-x_{n}\bigr)-(1- \alpha_{n})\omega_{n}AJx_{n},j \bigl(x_{n+1}-x^{*}\bigr)\bigr\rangle \\ =& \bigl\Vert x_{n}-x^{*} \bigr\Vert ^{2}+2\bigl\langle \alpha_{n}\bigl(f(x_{n})-x_{n}\bigr)-(1- \alpha_{n})\omega_{n}AJx_{n} \\ &{}+\alpha_{n}\bigl(x_{n+1}-x^{*}\bigr)-\alpha_{n} \bigl(x_{n+1}-x^{*}\bigr),j\bigl(x_{n+1}-x^{*}\bigr)\bigr\rangle \\ =& \bigl\Vert x_{n}-x^{*} \bigr\Vert ^{2}-2 \alpha_{n} \bigl\Vert x_{n+1}-x^{*} \bigr\Vert ^{2}+2\bigl\langle \alpha _{n}\bigl(f(x_{n})-x_{n} \bigr) \\ &{}-(1-\alpha_{n})\omega_{n}AJx_{n} +\alpha _{n} \bigl(x_{n+1}-x^{*}\bigr),j\bigl(x_{n+1}-x^{*}\bigr)\bigr\rangle \\ =& \bigl\Vert x_{n}-x^{*} \bigr\Vert ^{2}-2 \alpha_{n} \bigl\Vert x_{n+1}-x^{*} \bigr\Vert ^{2}+2\bigl\langle \alpha _{n}\bigl(f(x_{n})-x^{*} \bigr)+\alpha_{n}(x_{n+1}-x_{n}) \\ &{}-(1-\alpha_{n})\omega_{n}AJx_{n},j \bigl(x_{n+1}-x^{*}\bigr)\bigr\rangle , \end{aligned}$$

that is,

$$\begin{aligned} \bigl\Vert x_{n+1}-x^{*} \bigr\Vert ^{2} \leq& \bigl\Vert x_{n}-x^{*} \bigr\Vert ^{2}-2\alpha_{n} \bigl\Vert x_{n+1}-x^{*} \bigr\Vert ^{2}+2\bigl\langle \alpha_{n}\bigl(f(x_{n})-x^{*}\bigr)+\alpha_{n}^{2} \bigl(f(x_{n})-x_{n}\bigr) \\ &{}-\alpha_{n}(1-\alpha_{n})\omega_{n}AJx_{n}-(1- \alpha_{n})\omega_{n}AJx_{n},j \bigl(x_{n+1}-x^{*}\bigr)\bigr\rangle \\ \leq& \bigl\Vert x_{n}-x^{*} \bigr\Vert ^{2}-2 \alpha_{n} \bigl\Vert x_{n+1}-x^{*} \bigr\Vert ^{2}+2\bigl\langle \alpha _{n}\bigl(f(x_{n})-x^{*} \bigr)+\alpha_{n}^{2}\bigl(f(x_{n})-x^{*}\bigr) \\ &{}- \alpha_{n}^{2}\bigl(x_{n}-x^{*}\bigr) - \bigl(1-\alpha_{n}^{2}\bigr)\omega_{n}AJx_{n},j \bigl(x_{n+1}-x^{*}\bigr)\bigr\rangle \\ \leq& \bigl\Vert x_{n}-x^{*} \bigr\Vert ^{2}-2 \alpha_{n} \bigl\Vert x_{n+1}-x^{*} \bigr\Vert ^{2}+2\bigl[2\alpha_{n} \bigl\Vert f(x_{n})-x^{*} \bigr\Vert + \alpha_{n}^{2} \bigl\Vert x_{n}-x^{*} \bigr\Vert \\ &{}+\bigl(1-\alpha_{n}^{2}\bigr)\omega_{n} \bigl\Vert AJx_{n}-AJx^{*} \bigr\Vert \bigr] \bigl\Vert x_{n+1}-x^{*} \bigr\Vert . \end{aligned}$$

Since $\|x_{n+1}-x^{*}\|>\|x_{n}-x^{*}\|$ and *A* is *L*-Lipschitz and *J* is $L_{*}$-Lipschitz continuous respectively, thus we get

$$\begin{aligned} \alpha_{n} \bigl\Vert x_{n+1}-x^{*} \bigr\Vert \leq2 \alpha_{n} \bigl\Vert f(x_{n})-x^{*} \bigr\Vert + \alpha_{n}^{2} \bigl\Vert x_{n}-x^{*} \bigr\Vert +2(1-\alpha_{n})\omega_{n}LL_{*} \bigl\Vert x_{n}-x^{*} \bigr\Vert . \end{aligned}$$

Furthermore,

$$\begin{aligned} \bigl\Vert x_{n+1}-x^{*} \bigr\Vert \leq&2 \bigl\Vert f(x_{n})-x^{*} \bigr\Vert +\alpha_{n} \bigl\Vert x_{n}-x^{*} \bigr\Vert +2(1-\alpha _{n}) \frac{\omega_{n}}{\alpha_{n}}LL_{*} \bigl\Vert x_{n}-x^{*} \bigr\Vert \\ \leq& 2\ast\frac{r}{6}+\frac{r}{3}+2\ast\frac{1}{6LL_{*}}LL_{*}r \leq r. \end{aligned}$$

This is contradiction. Consequently, we can get that $\{x_{n}\}$ belongs to *B* for all integers $n\geq N_{0}$, which implies that the sequence $\{x_{n}\}$ is bounded, so are the sequences $\{f(x_{n})\}$ and $\{AJx_{n}\}$.

Moreover, it is easy to see that $\|x_{n+1}-x_{n}\|\rightarrow0$ because $\alpha_{n}\rightarrow0$ and $\omega_{n}=o(\alpha_{n})$,

$$\begin{aligned} \Vert x_{n+1}-x_{n} \Vert \leq\alpha_{n} \bigl\Vert f(x_{n})-x_{n} \bigr\Vert +(1- \alpha_{n})\omega_{n} \Vert AJx_{n} \Vert \rightarrow0. \end{aligned}$$

*Step 2*: We show that $\lim_{n\rightarrow\infty}\sup\langle z-f(x_{n}),j(z-x_{n+1})\rangle \leq0$, where $z\in C_{\min}\cap(AJ)^{-1}(0)$.

Since the sequences $\{x_{n}\}$ and $\{f(x_{n})\}$ are bounded, there exists $R>0$ sufficiently large such that $f(x_{n})$, $x_{n}\in B_{1}:=\overline{B_{R}(z)}$, $\forall n\in\mathbb{N}$. Furthermore, the set $B_{1}$ is a bounded closed and convex nonempty subset of *E*. By the convexity of $B_{1}$, we have that $(1-t)z+tf(x_{n})\in B_{1}$. Then it follows from the definition of *φ* that $\varphi(z)\leq\varphi((1-t)z+tf(x_{n}))$. Using Lemma 2.3, we have that

$$\begin{aligned} \bigl\Vert x_{n}-z-t\bigl(f(x_{n})-z\bigr) \bigr\Vert ^{2}\leq \Vert x_{n}-z \Vert ^{2}-2t\bigl\langle f(x_{n})-z,j\bigl(x_{n}-z-t\bigl(f(x_{n})-z \bigr)\bigr)\bigr\rangle , \end{aligned}$$

thus taking Banach limit over $n\geq1$,

$$\begin{aligned} \mu \bigl\Vert x_{n}-z-t\bigl(f(x_{n})-z\bigr) \bigr\Vert ^{2}\leq\mu \Vert x_{n}-z \Vert ^{2}-2t \mu\bigl\langle f(x_{n})-z,j\bigl(x_{n}-z-t \bigl(f(x_{n})-z\bigr)\bigr)\bigr\rangle , \end{aligned}$$

which means that

$$\begin{aligned} 2t\mu\bigl\langle f(x_{n})-z,j\bigl(x_{n}-z-t \bigl(f(x_{n})-z\bigr)\bigr)\bigr\rangle \leq&\mu \Vert x_{n}-z \Vert ^{2}-\mu \bigl\Vert x_{n}-z-t \bigl(f(x_{n})-z\bigr) \bigr\Vert ^{2} \\ =&\varphi(z)-\varphi\bigl(z+t\bigl(f(x_{n})-z\bigr)\bigr)\leq 0, \end{aligned}$$

that is,

$$\begin{aligned} \mu\bigl\langle f(x_{n})-z,j\bigl(x_{n}-z-t \bigl(f(x_{n})-z\bigr)\bigr)\bigr\rangle \leq0. \end{aligned}$$

By using the weak lower semi-continuity of the norm on *E*, we get the following as $t\rightarrow0$:

$$\begin{aligned} \bigl\langle f(x_{n})-z,j(x_{n}-z)\bigr\rangle -\bigl\langle f(x_{n})-z,j\bigl(x_{n}-z-t\bigl(f(x_{n})-z \bigr)\bigr)\bigr\rangle \rightarrow0. \end{aligned}$$

Thus, for $\forall\varepsilon> 0$, there exists $\delta>0$ such that $t \in(0,\delta)$, $n\geq1$

$$\begin{aligned} \bigl\langle f(x_{n})-z,j(x_{n}-z)\bigr\rangle < \bigl\langle f(x_{n})-z,j\bigl(x_{n}-z-t\bigl(f(x_{n})-z \bigr)\bigr)\bigr\rangle +\varepsilon, \end{aligned}$$

therefore,

$$\begin{aligned} \mu\bigl\langle f(x_{n})-z,j(x_{n}-z)\bigr\rangle < \mu\bigl\langle f(x_{n})-z,j\bigl(x_{n}-z-t\bigl(f(x_{n})-z \bigr)\bigr)\bigr\rangle +\varepsilon. \end{aligned}$$

In view of the arbitrariness of *ε*, we have that

$$\begin{aligned} \mu\bigl\langle f(x_{n})-z,j(x_{n}-z)\bigr\rangle \leq0. \end{aligned}$$

From the norm-to-weak* uniform continuity of *J* on each bounded subset of *E*, we have that

$$\begin{aligned} \lim_{n\rightarrow\infty}\bigl(\bigl\langle f(x_{n})-z,j(x_{n+1}-z) \bigr\rangle -\bigl\langle f(x_{n})-z,j(x_{n}-z)\bigr\rangle \bigr)=0. \end{aligned}$$

Thus, the sequence $\{\langle f(x_{n})-z,j(x_{n}-z)\rangle\}$ satisfies the condition of Lemma 2.1, so we have that

3.4 $$ \limsup_{n\rightarrow\infty}\bigl\langle f(x_{n})-z,j(x_{n+1}-z) \bigr\rangle \leq0. $$

*Step 3*: Next we show that $\|x_{n+1}-z\| \rightarrow 0$.

From (3.2), (3.3) and Lemma 2.3 we have that

$$\begin{aligned} \Vert x_{n+1}-z \Vert ^{2} =& \Vert x_{n+1}-x_{n}+x_{n}-z \Vert ^{2} \\ =& \bigl\Vert x_{n}-z+\alpha_{n}\bigl(f(x_{n})-x_{n} \bigr)-(1-\alpha_{n})\omega_{n}AJx_{n} \bigr\Vert ^{2} \\ =& \bigl\Vert (1-\alpha_{n}) (x_{n}-z)+ \alpha_{n}\bigl(f(x_{n})-z\bigr)-(1-\alpha_{n}) \omega_{n}AJx_{n} \bigr\Vert ^{2} \\ \leq& (1-\alpha_{n})^{2} \Vert x_{n}-z \Vert ^{2}+2\bigl\langle \alpha_{n}\bigl(f(x_{n})-z \bigr)-(1-\alpha_{n})\omega_{n}AJx_{n},j(x_{n+1}-z) \bigr\rangle . \end{aligned}$$

In view of the fact that the sequence $\{x_{n}\}$ is bounded, without loss of generality, we assume that $M:=\sup\{\|x_{n}-z\|\}$, therefore,

$$\begin{aligned} \Vert x_{n+1}-z \Vert ^{2} \leq& (1-\alpha_{n})^{2} \Vert x_{n}-z \Vert ^{2}+2\bigl\langle \alpha _{n}\bigl(f(x_{n})-z\bigr)-(1-\alpha_{n}) \omega_{n}AJx_{n},j(x_{n+1}-z)\bigr\rangle \\ =& (1-\alpha_{n}) \Vert x_{n}-z \Vert ^{2}+2\bigl\langle \alpha_{n}\bigl(f(x_{n})-z \bigr), j(x_{n+1}-z)\bigr\rangle \\ &{}+2(1-\alpha_{n})\omega_{n} \Vert AJz-AJx_{n} \Vert \Vert x_{n+1}-z \Vert \\ \leq&(1-\alpha_{n}) \Vert x_{n}-z \Vert ^{2}+\sigma_{n}, \end{aligned}$$

where $\sigma_{n}=2\alpha_{n} \langle(f(x_{n})-z),j(x_{n+1}-z)\rangle +2\omega_{n}LL_{*}M^{2}$.

From Lemma 2.2 and (3.4) we shall obtain that

$$\begin{aligned} \lim_{n\rightarrow\infty} \Vert x_{n}-z \Vert =0, \end{aligned}$$

which means that the consequence $\{x_{n}\}$ converges strongly to *z*. The proof is complete. □

### Theorem 3.3

*Let* *E* *be a uniformly convex and* 2-*uniformly smooth Banach space*. *Assume that* $A:E^{*}\rightarrow E$ *is an* *L*-*Lipschitz continuous monotone mapping such that* $C_{\min}\cap (AJ)^{-1}(0) \neq\emptyset$. *Then*, *for any* $x_{0}\in E$, *the sequence* $\{x_{n}\}$ *defined by*

3.5 $$ x_{n+1}=\alpha_{n}x_{n}+(1-\alpha_{n}) (I-\omega_{n}AJ)x_{n} $$

*converges strongly to an element* $z\in(AJ)^{-1}(0)$.

### Proof

Similar to the proof in Theorem 3.2, we can obtain that the sequences $\{x_{n}\}$ and $\{AJx_{n}\}$ are bounded. Furthermore, we have that $\lim_{n\rightarrow\infty}\sup\langle x_{n}-z,j(x_{n+1}-z)\rangle\leq0$, where $z\in C_{\min}\cap (AJ)^{-1}(0) $.

In addition, the recurrence (3.5) can be rewritten as

$$\begin{aligned} x_{n+1}=x_{n}-(1-\alpha_{n})\omega_{n}AJx_{n}. \end{aligned}$$

It is easy to see that $\|x_{n+1}-x_{n}\|=(1-\alpha_{n})\omega_{n}\|AJx_{n}\| \rightarrow0$ as $\alpha_{n}\rightarrow0$.

From the recursion (3.5) and Lemma 2.4 we have that

$$\begin{aligned} \Vert x_{n+1}-z \Vert ^{2} =& \bigl\Vert (1-\alpha_{n}) (x_{n}-z)+\alpha_{n}(x_{n}-z)-(1- \alpha _{n})\omega_{n}AJx_{n} \bigr\Vert ^{2} \\ \leq& (1-\alpha_{n})^{2} \Vert x_{n}-z \Vert ^{2}+2\alpha_{n}\bigl\langle x_{n}-z,j(x_{n+1}-z) \bigr\rangle \\ &{}-2(1-\alpha_{n})\omega_{n}\bigl\langle AJx_{n},j(x_{n+1}-z)\bigr\rangle \\ \leq& (1-\alpha_{n}) \Vert x_{n}-z \Vert ^{2}+2\alpha_{n}\bigl\langle x_{n}-z,j(x_{n+1}-z) \bigr\rangle +2(1-\alpha_{n})\omega_{n}LL_{*}M^{2} \\ \leq& (1-\alpha_{n}) \Vert x_{n}-z \Vert ^{2}+2\alpha_{n}\bigl\langle x_{n}-z,j(x_{n+1}-z) \bigr\rangle +2\omega_{n}LL_{*}M^{2}, \end{aligned}$$

where $M:=\sup\{\|x_{n}-z\|\}$. It follows from Lemma 2.2 that $\lim_{n\rightarrow\infty}\|x_{n}-z\|=0$, which means that the sequence $\{x_{n}\}$ converges strongly to an element $z\in(AJ)^{-1}(0)$. The proof is complete. □

According to Zegeye [1] and Liu [32], for a mapping $T:E\rightarrow E^{*}$, a point $x^{*}\in E$ is called a *J*-fixed point of *T* if and only if $Tx^{*}=Jx^{*}$ and *T* is called semi-pseudo if and only if $A:=J-T$ is monotone. We can observe that a zero point of *A* is the *J*-fixed point of a semi-pseudo mapping. If *E* is a Hilbert space, the semi-pseudo mapping and the *J*-fixed point coincide with a pseudo-contractive mapping and a fixed point of pseudo-contraction, respectively. In the case that the semi-pseudo mapping *T* is from $E^{*}$ to *E*, we have that $AJ:=(J^{-1}-T)J$ is monotone and the *J*-fixed point set is denoted by $F_{J}(T)=\{x\in E, x=TJx\}$. We have the following corollaries for semi-pseudo mappings from $E^{*}$ to *E*.

### Corollary 3.4

*Let* *E* *be a uniformly convex and* 2-*uniformly smooth Banach space*. *Assume that* $T:E^{*}\rightarrow E$ *is an* *L*-*Lipschitz continuous semi*-*pseudo mapping such that* $C_{\min}\cap F_{J}(T)\neq\emptyset$ *and* $f:E\rightarrow E$ *is a piecewise function defined as* (C2). *Then*, *for any* $x_{0}\in E$, *the sequence* $\{x_{n}\}$ *defined by*

$$ x_{n+1}=\alpha_{n}f(x_{n})+(1- \alpha_{n}) \bigl((1-\omega_{n})I+\omega_{n}TJ \bigr)x_{n} $$

*converges strongly to an element* $z\in F_{J}(T)$.

### Corollary 3.5

(Zegeye [1])

*Let* *E* *be a uniformly convex and* 2-*uniformly smooth Banach space*. *Assume that* $A:E^{*}\rightarrow E$ *is an* *L*-*Lipschitz continuous monotone mapping such that* $C_{\min}\cap (AJ)^{-1}(0) \neq\emptyset$. *Then*, *for any* $u\in E$, *the sequence* $x_{n}$ *defined by*

$$ x_{n+1}=\alpha_{n}u+(1-\alpha_{n}) (I- \omega_{n}AJ)x_{n} $$

*converges strongly to an element* $z\in(AJ)^{-1}(0)$.

### Proof

Take $f(x)\equiv u$ in Theorem 3.2, the result is obtained. □

If we change the role of *E* and $E^{*}$, then we shall obtain the following results.

### Theorem 3.6

*Let* *E* *be a uniformly convex and* 2-*uniformly smooth Banach space*. *Assume that* $A:E\rightarrow E^{*}$ *is an* *L*-*Lipschitz continuous monotone mapping such that* $C_{\min}\cap(AJ^{-1})^{-1}(0)\neq\emptyset$. *Then*, *for any* $x_{0}\in E$, *the sequence* $\{x_{n}\}$ *defined by*

$$\begin{aligned} x_{n+1}=J^{-1}\bigl(\alpha_{n}Jx_{n}+(1- \alpha_{n}) (J-\omega_{n}A)x_{n}\bigr),\quad n\geq1, \end{aligned}$$

*converges strongly to an element* $z\in(AJ^{-1})^{-1}(0)$.

### Theorem 3.7

(Zegeye [1])

*Let* *E* *be a uniformly convex and* 2-*uniformly smooth Banach space*. *Assume that* $A:E\rightarrow E^{*}$ *is an* *L*-*Lipschitz continuous monotone mapping such that* $C_{\min}\cap(AJ^{-1})^{-1}(0)\neq\emptyset$. *Then*, *for any* $u\in E$, *the sequence* $\{x_{n}\}$ *defined by*

$$\begin{aligned} x_{n+1}=J^{-1}\bigl(\alpha_{n}Ju+(1- \alpha_{n}) (J-\omega_{n}A)x_{n}\bigr),\quad n\geq1, \end{aligned}$$

*converges strongly to an element* $z\in(AJ^{-1})^{-1}(0)$.

We give below two examples in order to show that the conditions of explicit iterative Algorithm (3.2) are easily satisfied.

### Example 1

We take the parameters as follows:

$$\begin{aligned} \alpha_{n}=\frac{1}{(n+1)^{p}},\qquad \omega_{n}= \frac{1}{n(n+1)^{p}},\quad (0< p\leq1). \end{aligned}$$

It is easy to verify that

1. $\lim_{n\rightarrow\infty}\alpha_{n}=0$, $\sum_{n=1}^{\infty}\alpha_{n}=\infty$;
2. $\lim_{n\rightarrow\infty}\frac{\omega_{n}}{\alpha_{n}}=0$ and $\sum_{n=1}^{\infty}\omega_{n}<\infty$.

### Example 2

We take the parameters as follows:

$$\begin{aligned} \alpha_{n}=\frac{1}{\ln^{p}(n+1)},\qquad \omega_{n}= \frac{1}{n\ln^{p}(n+1)},\quad (0< p\leq1). \end{aligned}$$

It is easy to verify that

1. $\lim_{n\rightarrow\infty}\alpha_{n}=0$, $\sum_{n=1}^{\infty}\alpha_{n}=\infty$;
2. $\lim_{n\rightarrow\infty}\frac{\omega_{n}}{\alpha_{n}}=0$ and $\sum_{n=1}^{\infty}\omega_{n}<\infty$.

## Applications

In this section, we consider the constrained convex minimization problems and the solution of Hammerstein integral equations as the applications of our main result which is proposed in Sect. 3.

### Application to constrained convex minimization problems

In this subsection, we will consider the following minimization problem:

4.1 $$\begin{aligned} \min_{x\in C}h(x), \end{aligned}$$

where *C* is a nonempty closed convex subset of *E*, and $h:C\rightarrow R$ is a real-valued convex function. Assume that problem (4.1) is consistent (i.e., its solution set is nonempty). According to Diop et al. [21], $x\in E$ is a minimizer of *h* if and only if $0 \in\partial h(x)$.

#### Lemma 4.1

*Let* *E* *be a real normed smooth space and* $h:E\rightarrow\mathbb{R}$ *be a differential convex function*. *Assume that the function* *h* *is bounded*, *then the sub*-*differential map* $\partial h:E\rightarrow\mathbb{R}$ *is bounded and the following inequality holds*:

$$\begin{aligned} \bigl\langle \partial h(x)-\partial h(y),x-y\bigr\rangle \geq\langle Jx-Jy,x-y \rangle, \quad \forall x,y\in E. \end{aligned}$$

#### Proof

Define $g:=h-\frac{1}{2} \Vert \cdot \Vert ^{2}$, then $h=g+\frac{1}{2} \Vert \cdot \Vert ^{2}$. Since *h* and $\Vert \cdot \Vert ^{2}$ are differential, so *g* is differential and the sub-differential of *g* is denoted by $\partial g=\partial h-J$. Let $x\in E$, we can get from the definition of *∂g* that

$$\begin{aligned} g(y)-g(x)\geq \bigl\langle y-x,\partial g(x) \bigr\rangle ,\quad \forall y\in E, \end{aligned}$$

which means that

4.2 $$\begin{aligned} h(y)-\frac{1}{2} \Vert y \Vert ^{2}-h(x)+\frac{1}{2} \Vert x \Vert ^{2}\geq \bigl\langle y-x,\partial h(x)-Jx \bigr\rangle , \quad \forall y\in E. \end{aligned}$$

Exchanging *x* and *y* in the above inequality (4.2), we have that

4.3 $$\begin{aligned} h(x)-\frac{1}{2} \Vert x \Vert ^{2}-h(y)+\frac{1}{2} \Vert y \Vert ^{2}\geq \bigl\langle x-y,\partial h(y)-Jy \bigr\rangle ,\quad \forall x\in E. \end{aligned}$$

Adding the above inequalities (4.2) and (4.3), we get that

$$\begin{aligned} \bigl\langle \partial h(x)-\partial h(y),x-y \bigr\rangle \geq \langle x-y,Jx-Jy \rangle. \end{aligned}$$

This completes the proof. □

#### Remark 4.2

From Lemma 4.1, the sub-differential *∂h* is monotone, we can also get that $T=J-\partial h$ is a semi-pseudo mapping from *E* to $E^{*}$.

Consequently, the following theorems are obtained.

#### Theorem 4.3

*Let* *E* *be a uniformly convex and* 2-*uniformly smooth real Banach space*. *Assume that* $h:E\rightarrow\mathbb{R}$ *is a proper*, *convex*, *bounded*, *and coercive function such that* $C_{\min}\cap (\partial hJ)^{-1}(0)\neq\emptyset$ *and* $f:E\rightarrow E$ *is a piecewise function defined as* (C2). *Then*, *for any* $x_{0}\in E$, *the sequence* $\{x_{n}\}$ *defined by*

$$\begin{aligned} x_{n+1}=\alpha_{n}f(x_{n})+(1- \alpha_{n}) (I-\omega_{n}\partial hJ)x_{n}, \quad n \geq1, \end{aligned}$$

*converges strongly to an element* $x^{*}\in(\partial h J)^{-1}(0)$, *that is*, $Jx^{*}\in(\partial h)^{-1}(0)$. *Then function* *h* *has a unique minimizer* $Jx^{*}\in E^{*}$ *and the sequence* $\{x_{n}\}$.

#### Theorem 4.4

*Let* *E* *be a uniformly convex and* 2-*uniformly smooth real Banach space*. *Assume that* $h:E\rightarrow\mathbb{R}$ *is a proper*, *convex*, *bounded*, *and coercive function such that* $C_{\min}\cap (\partial hJ)^{-1}(0)\neq\emptyset$. *Then*, *for any* $x_{0}\in E$, *the sequence* $\{x_{n}\}$ *defined by*

$$\begin{aligned} x_{n+1}=\alpha_{n}x_{n}+(1-\alpha_{n}) (I-\omega_{n}\partial hJ),\quad n\geq1, \end{aligned}$$

*converges strongly to an element* $x^{*}\in(\partial h J)^{-1}(0)$, *that is*, $Jx^{*}\in (\partial h)^{-1}(0)$.

### Application to solution of Hammerstein integral equations

An integral equation (generally nonlinear) of Hammerstein type has the form

4.4 $$\begin{aligned} u(x)+ \int_{\Omega}k(x,y)f\bigl(y,u(y)\bigr)=w(x), \end{aligned}$$

where the unknown function *u* and the inhomogeneous function *w* lie in a Banach space *E* of measurable real-valued functions.

By simple transformation, (4.4) shall be written as

$$\begin{aligned} u+KFu=w, \end{aligned}$$

which can be illustrated, without loss of generality, as

4.5 $$\begin{aligned} u+KFu=0. \end{aligned}$$

For the case of a real Hilbert space *H*, for $F, K:H\rightarrow H$, Chidume and Zegeye [33] defined an auxiliary map on the Cartesian product $E:=H\times H$, $T:E\rightarrow E$ by

$$\begin{aligned} T[u,v]=[Fu-v,Kv+u]. \end{aligned}$$

It is known that

$$T[u,v]=0\quad \Leftrightarrow\quad u \mbox{ is the solution of (4.5) and }v=Fu. $$

They obtained strong convergence of an iterative algorithm defined in the Cartesian product space *E* to a solution of Hammerstein Eq. (4.5).

In a Banach space more general than a Hilbert space, Zegeye [34], Chidume and Idu [35] introduced the operator $T:E\times E^{*}\rightarrow E^{*}\times E$:

$$\begin{aligned} T[u,v]=[Ju-Fu+v,J_{*}v-Kv-u], \end{aligned}$$

where $F:E\rightarrow E^{*}$ and $K:E^{*}\rightarrow E$ are monotone mappings and $J_{*}$ is the normalized duality map from $E^{*}$ to *E*. They proved that the mapping $A:=J-T$ defined by $A[u,v]:=[Fu-v, Kv+u]$ is monotone and $u^{*}$ is a solution (when they exist) of the Hammerstein equation $u+KFu=0$ if and only if $(u^{*},v^{*})$ is a zero point of *A*, where $v^{*}=Fu^{*}$. Applying our Theorem 3.2, the following theorems shall be obtained.

#### Theorem 4.5

*Let* *E* *be a uniformly convex and* 2-*uniformly smooth Banach space*. *Assume that* $F:E\rightarrow E^{*}$, $K:E^{*}\rightarrow E$ *are Lipschitz continuous monotone mappings such that Hammerstein Eq*. (4.5) *is solvable and* $f_{1}:E\rightarrow E$, $f_{2}:E^{*}\rightarrow E^{*}$ *are two piecewise functions defined as* (C2). *Then*, *for* $(u_{0},v_{0})\in E\times E^{*}$, *the sequences* $\{u_{n}\}$ *and* $\{v_{n}\}$ *defined by*

$$ \begin{aligned} &u_{n+1}=\alpha_{n}f_{1}(u_{n})+(1- \alpha_{n}) \bigl(u_{n}-\omega_{n}J_{*}(Fu_{n}-v_{n}) \bigr), \\ &v_{n+1}=\alpha_{n}f_{2}(v_{n})+(1- \alpha_{n}) \bigl(v_{n}-\omega_{n}J(Kv_{n}+u_{n}) \bigr), \end{aligned} $$

*converge strongly to* $u^{*}$ *and* $v^{*}$, *respectively*, *where* $u^{*}$ *is a solution of* $u+KFu=0$ *with* $v^{*}=Fu^{*}$.

#### Theorem 4.6

*Let* *E* *be a uniformly convex and* 2-*uniformly smooth Banach space*. *Assume that* $F:E\rightarrow E^{*}$, $K:E^{*}\rightarrow E$ *are Lipschitz continuous monotone mappings such that Hammerstein Eq*. (4.5) *is solvable*. *Then*, *for* $(u_{0},v_{0})\in E\times E^{*}$, *the sequences* $\{u_{n}\}$ *and* $\{v_{n}\}$, *defined by*

$$ \begin{aligned} &u_{n+1}=\alpha_{n}u_{n}+(1-\alpha_{n}) \bigl(u_{n}-\omega_{n}J_{*}(Fu_{n}-v_{n}) \bigr), \\ &v_{n+1}=\alpha_{n}v_{n}+(1-\alpha_{n}) \bigl(v_{n}-\omega_{n}J(Kv_{n}+u_{n}) \bigr), \end{aligned} $$

*converge strongly to* $u^{*}$ *and* $v^{*}$, *respectively*, *where* $u^{*}$ *is a solution of* $u+KFu=0$ *with* $v^{*}=Fu^{*}$.

## Numerical example

In the sequel, we give a numerical example to illustrate the applicability, effectiveness, efficiency, and stability of our viscosity iterative algorithm (VIA). We have written all the codes in Matlab R2016b and they are preformed on a LG dual core personal computer.

### Numerical behavior of VIA

#### Example

Let $E=\mathbb{R}$, $C=E$. Let $A, J:\mathbb {R}\rightarrow\mathbb{R}$ be the mappings defined as

$$ Ax=ax,\qquad Jx=x, $$

$f:C\rightarrow C$ be defined as

$$ f(x)= \textstyle\begin{cases} \frac{x}{2}, &Ax\neq0,\\ x,& \mbox{if }Ax=0. \end{cases} $$

Thus, for $x,y\in\mathbb{R}$, we have

$$\begin{aligned}& \begin{aligned} \Vert Ax-Ay \Vert &= \Vert ax-ay \Vert \\ &\leq \vert a \vert \ast \Vert x-y \Vert , \end{aligned} \\& \Vert Jx-Jy \Vert = \Vert x-y \Vert . \end{aligned}$$

Hence, *A* is $|a|$-Lipschitz continuous monotone, *J* is 1-Lipschitz continuous.

Two groups of consequences of parameters are tested here as follows:

Case I: $\alpha_{n}=\frac{1}{(n+1)^{p}}$, $\omega_{n}=\frac{1}{n(n+1)^{p}}$, $p\in[1/8,1/4,1/3,1/2,1]$;

Case II: $\alpha_{n}=\frac{1}{\ln^{p}(n+1)}$, $\omega_{n}=\frac{1}{n\ln ^{p}(n+1)}$, $p\in[1/8,1/4,1/3,1/2,1]$.

We can see that all these parameters satisfy the conditions:

(i) $\lim_{n\rightarrow\infty}\alpha_{n}=0$, $\sum_{n=1}^{\infty}\alpha_{n}=\infty$;
(ii) $\omega_{n}=o(\alpha_{n})$, $\sum_{n=1}^{\infty}\omega_{n}<\infty$.

We will use the sequence $D_{n}=10^{8}\times\|x_{n+1}-x_{n}\|^{2}$ to study the convergence of our explicit viscosity iterative algorithm (VIA). The convergence of $D_{n}$ to 0 implies that the sequence $\{x_{n}\}$ converges to $x^{*}\in(AJ)^{-1}(0)$. To illustrate the behavior of the algorithm, we have performed experiments for both number of iterations (iter.) and elapsed execution time (CPU time—in the second). Figures 1–18 and Table 1 describe the behavior of $D_{n}$ generated by VIA for the aforementioned groups of parameters. It is obvious that if $x_{n}\in(AJ)^{-1}(0)$, then the process stops and $x_{n}$ is the solution of problem $0\in AJu$; otherwise, we shall compute the following viscosity algorithm:

$$ x_{n+1}= \frac{\alpha_{n}x_{n}}{2}+(1-\alpha_{n}) (x_{n}-a\omega_{n}x_{n}), $$

where *a* is different choices from $[\frac{1}{100}, \frac{1}{2}, 2]$.

**Figure 1:**
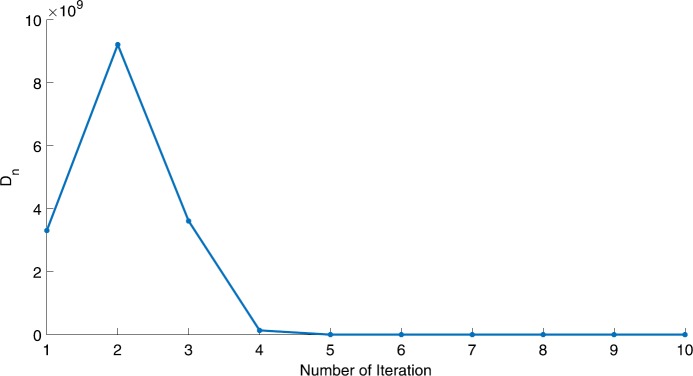
Case I, $a=\frac{1}{100}$, $p=\frac{1}{2}$

**Figure 2:**
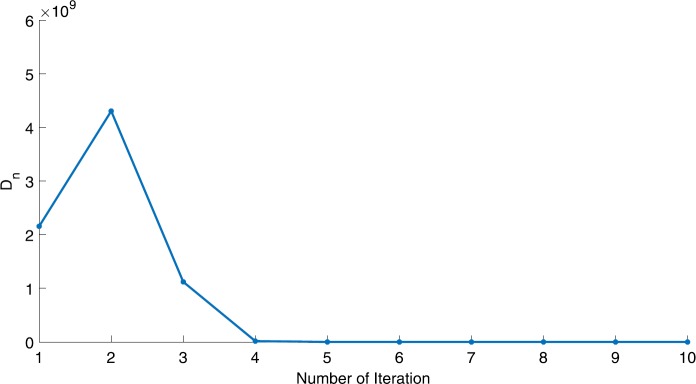
Case I, $a=\frac{1}{100}$, $p=\frac{1}{3}$

**Figure 3:**
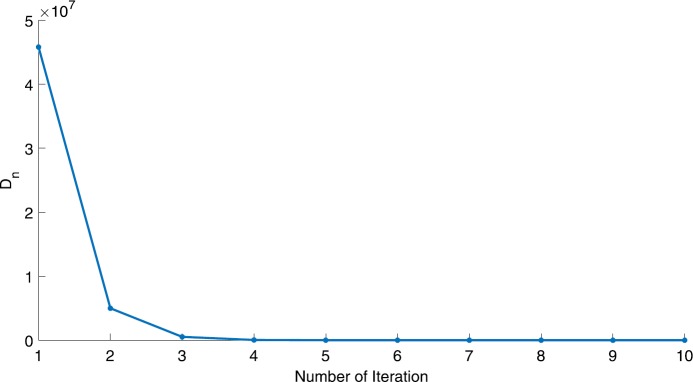
Case I, $a=\frac{1}{2}$, $p=\frac{1}{2}$

**Figure 4:**
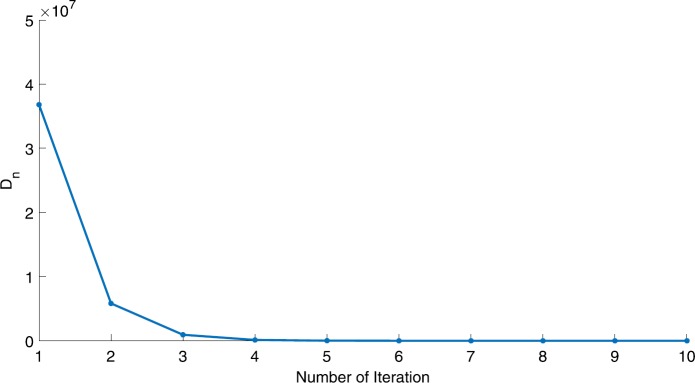
Case I, $a=\frac{1}{2}$, $p=\frac{1}{4}$

**Figure 5:**
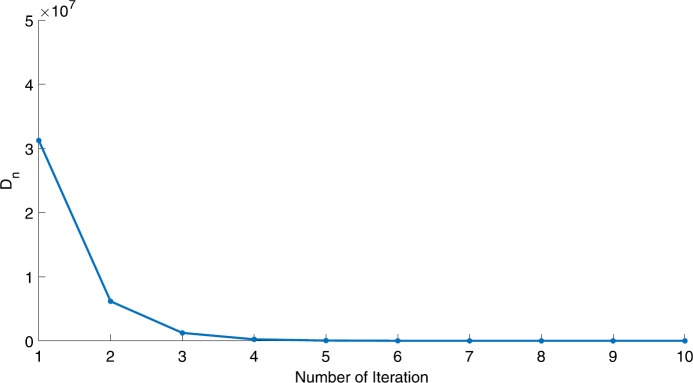
Case I, $a=\frac{1}{2}$, $p=\frac{1}{8}$

**Figure 6:**
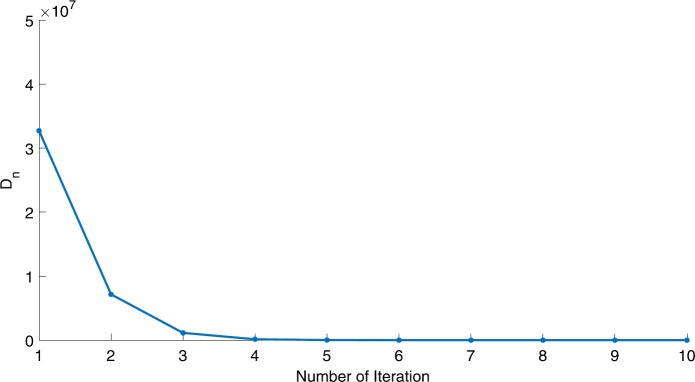
Case I, $a=2$, $p=\frac{1}{3}$

**Figure 7:**
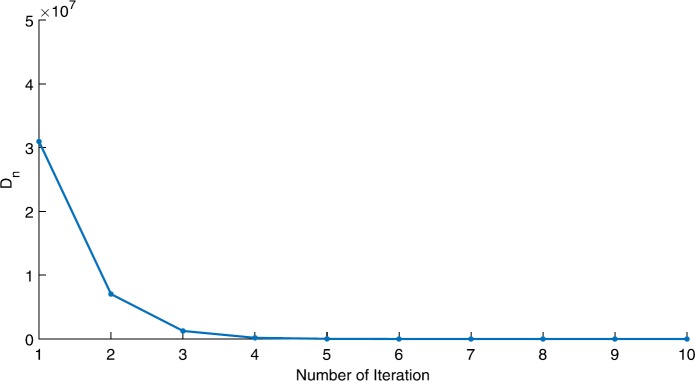
Case I, $a=2$, $p=\frac{1}{4}$

**Figure 8:**
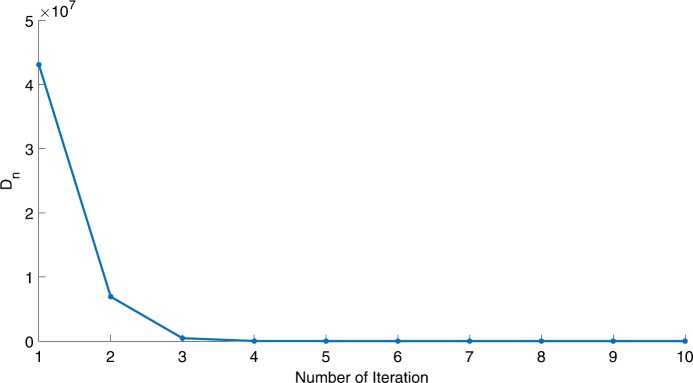
Case I, $a=2$, $p=2$

**Figure 9:**
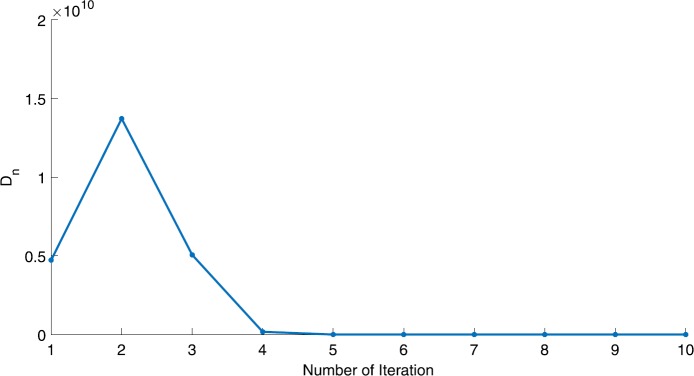
Case I, $a=\frac{1}{100}$, $p=2$

**Figure 10:**
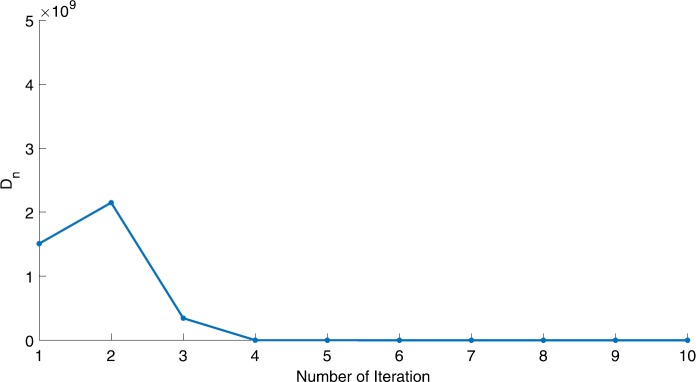
Case I, $a=\frac{1}{100}$, $p=\frac{1}{4}$

**Figure 11:**
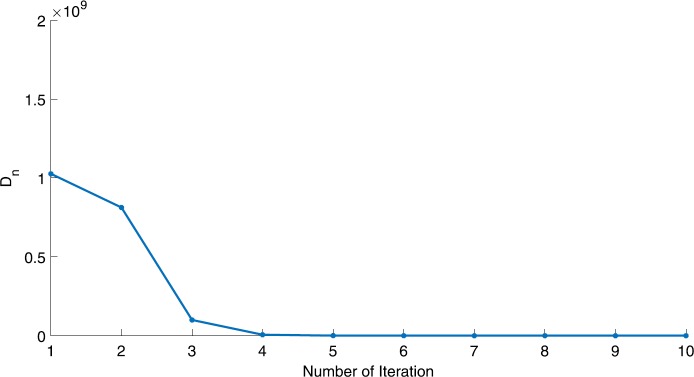
Case II, $a=\frac{1}{100}$, $p=\frac{1}{3}$

**Figure 12:**
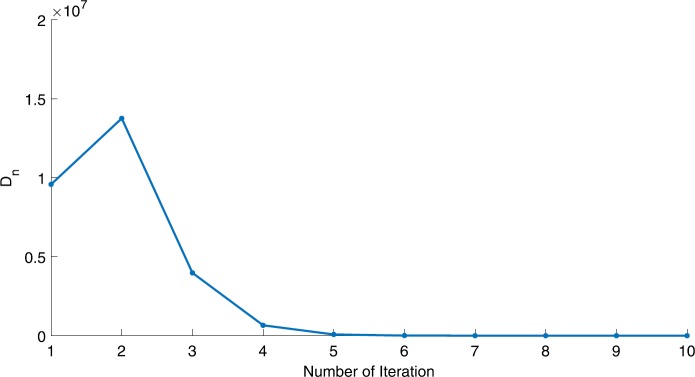
Case II, $a=2$, $p=1$

**Figure 13:**
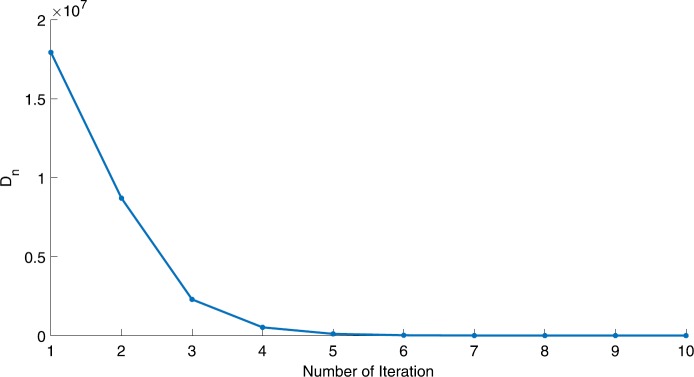
Case II, $a=\frac{1}{2}$, $p=\frac{1}{4}$

**Figure 14:**
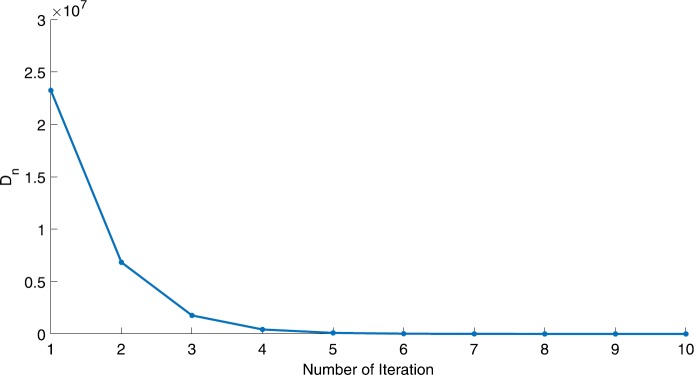
Case II, $a=2$, $p=\frac{1}{8}$

**Figure 15:**
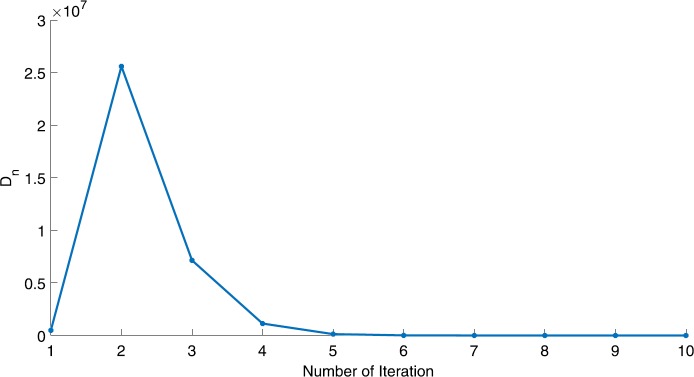
Case II, $a=\frac{1}{2}$, $p=1$

**Figure 16:**
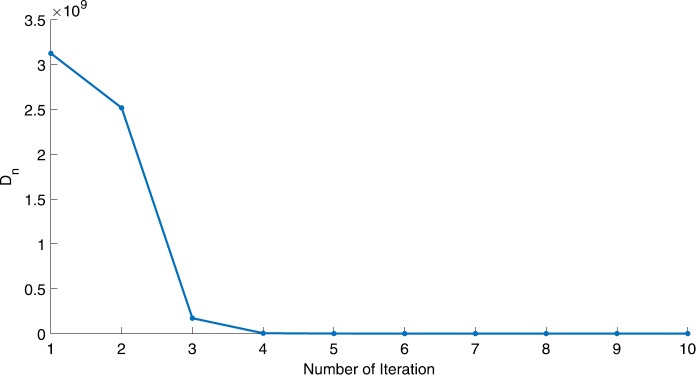
Case II, $a=\frac{1}{100}$, $p=\frac{1}{2}$

**Figure 17:**
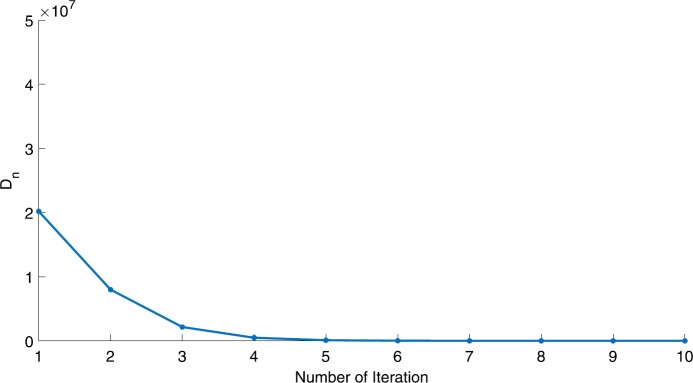
Case II, $a=2$, $p=\frac{1}{3}$

**Figure 18:**
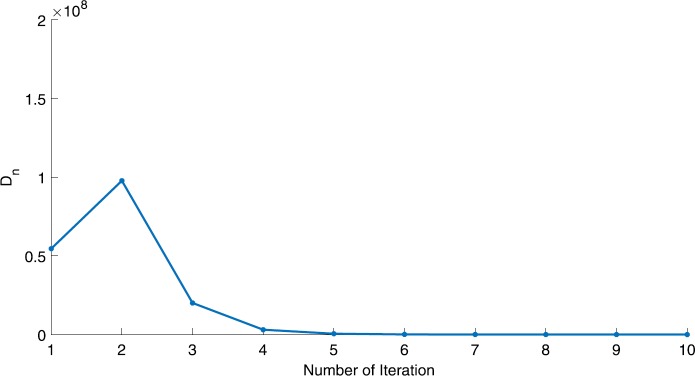
Case II, $a=\frac{1}{100}$, $p=\frac{1}{8}$

**Table 1:** Algorithm (VIA) with different group of parameters

	*a*	0.01	0.5	0.99
*p* = 1				
Case I	No. Iterations	10	10	12
	CPU (time)	0.047	0.047	0.055
Case II	No. Iterations	10	10	12
	CPU (time)	0.044	0.048	0.045
*p* = 2				
Case I	No. of Iterations	10	10	12
	CPU (time)	0.056	0.058	0.062
Case II	No. Iterations	10	10	12
	CPU (time)	0.068	0.055	0.052

In these figures, *x*-axes represent the number of iterations while *y*-axes represent the value of $D_{n}$. We can summarize the following observations from these figures:

- The rate of $D_{n}=10^{10}\times\|x_{n+1}-x_{n}\|^{2}$ generated by our algorithm (VIA) depends strictly on the convergence rate of parameter $\{\alpha_{n}\}$ and the Lipschitz coefficient of a continuous monotone operator.
- Our viscosity iterative algorithm (VIA) works well for parameter sequences of $\{\alpha_{n}\}$ being fast convergent to 0 as $n\rightarrow\infty$. In general, if $D_{n}=\| x_{n+1}-x_{n}\|^{2}$, then the error of $D_{n}$ can be obtained approximately equal to 10^−16^. When $D_{n}$ obtains to this error, then it becomes unstable. The best error of $D_{n}$ can be obtained approximately equal to 10^−30^ when $a=2$.
- For the second group parameter $\{\alpha_{n}\}$ being slowly convergent to 0 as $n\rightarrow\infty$, then $D_{n}$ is slightly increasing in the early iterations, and after that, it is seen to be almost stable.

### Comparison of VIA with other algorithms

In this part, we present several experiments in comparison with other algorithms. Two methods used in comparison are the generalized Mann iteration method (GMIM) (Chidume et al. [35], Algorithm 1) and the regularization method (RM) (Zegeye [1], Algorithm 2). The RM requires to previously know a constant *u*. For experiments, we choose the same sequences $\alpha _{n}=\frac{1}{n+1}$ and $\omega_{n}=\frac{1}{n(n+1)}$ in these algorithms. The condition $\|x_{n+1}-x_{n}\|^{2}\leq TOL$ is chosen to be as the stopping criterion. The following tables are comparisons of VIA, RM, GMIM with different choices of *a*. The numerical results are showed in Table 2.

**Table 2:** Comparison between VIA and other algorithms with $x_{0}=1$

*a*	TOL	VIA		RM (*u* = 1)		GMIM	
		Iter	CPU (s)	Iter	CPU (s)	Iter	CPU (s)
1/4	10^−8^	278	0.094	85	0.055	559	0.097
	10^−10^	1290	0.14	325	0.070	3529	0.28
1/3	10^−8^	266	0.076	100	0.044	473	0.078
	10^−10^	1233	0.14	380	0.088	2663	0.21
1/2	10^−8^	243	0.070	126	0.052	317	0.065
	10^−10^	1123	0.13	472	0.077	1471	0.13
3/2	10^−8^	128	0.061	221	0.059	37	0.043
	10^−10^	587	0.10	823	0.098	94	0.049

From these tables, we can see that the RM is the best. The GMIM is the most time-consuming, and the reasonable explanation is the fact that at each step the GMIM has no contractive parameters (coefficients) for obtaining the next step which can take lower convergence rate, while the convergence rate of the RM depends strictly on the previous constant *u* and the initial value $x_{0}$. In comparing with other two methods, VIA seems to have competitive advantage. However, the main advantage of VIA is that the viscosity iterative algorithm works more stable than other methods and it is done in Banach spaces much more general than Hilbert spaces.

## Conclusion

Let *E* be a nonempty closed uniformly convex and 2-uniformly smooth Banach space with dual $E^{*}$. We construct some implicit and explicit algorithms for solving the equation $0\in AJu$ in the Banach space *E*, where $A:E^{*}\rightarrow E$ is a monotone mapping and $J:E\rightarrow E^{*}$ is the normalized duality map which plays an indispensable role in this research paper. The advantages of the algorithm are that the resolvent operator is not involved, which makes the iteration simple for computation; moreover, the zero point problem of monotone mappings is extended from Hilbert spaces to Banach spaces. The proposed algorithms converge strongly to a zero of the composed mapping *AJ* under concise parameter conditions. In addition, the main result is applied to approximate the minimizer of a proper convex function and the solution of Hammerstein integral equations. To some extent, our results extend and unify some results considered in Xu [12], Zegeye [1], Chidume and Idu [2], Chidume [3, 35], and Ibarakia and Takahashi [22].
